# 3D-QSPR Method of Computational Technique Applied on Red Reactive Dyes by Using CoMFA Strategy

**DOI:** 10.3390/ijms12128862

**Published:** 2011-12-05

**Authors:** Uzma Mahmood, Sitara Rashid, S. Ishrat Ali, Rasheeda Parveen, Zaheer-ul-Haq, Nida Ambreen, Khalid Mohammed Khan, Shahnaz Perveen, Wolfgang Voelter

**Affiliations:** 1 Dr. Panjwani Center for Molecular Medicine and Drug Research, International Center for Chemical and Biological Sciences, University of Karachi, Karachi 75270, Pakistan; 2 Department of Applied Chemistry, University of Karachi, Karachi 75270, Pakistan; 3 H. E. J. Research Institute of Chemistry, International Center for Chemical and Biological Sciences, University of Karachi, Karachi 75270, Pakistan; 4 PCSIR Laboratories Complex, Karachi, Shahrah-e-Dr. Salimuzzaman Siddiqui, Karachi 75280, Pakistan; 5 Interfakultäres Institut für Biochemie der Universität Tübingen, Hoppe-Seyler Straße 4, Tübingen D-72076, Germany

**Keywords:** 3D-QSPR, CoMFA, red reactive dye, cellulose fiber

## Abstract

Cellulose fiber is a tremendous natural resource that has broad application in various productions including the textile industry. The dyes, which are commonly used for cellulose printing, are “reactive dyes” because of their high wet fastness and brilliant colors. The interaction of various dyes with the cellulose fiber depends upon the physiochemical properties that are governed by specific features of the dye molecule. The binding pattern of the reactive dye with cellulose fiber is called the ligand-receptor concept. In the current study, the three dimensional quantitative structure property relationship (3D-QSPR) technique was applied to understand the red reactive dyes interactions with the cellulose by the Comparative Molecular Field Analysis (CoMFA) method. This method was successfully utilized to predict a reliable model. The predicted model gives satisfactory statistical results and in the light of these, it was further analyzed. Additionally, the graphical outcomes (contour maps) help us to understand the modification pattern and to correlate the structural changes with respect to the absorptivity. Furthermore, the final selected model has potential to assist in understanding the charachteristics of the external test set. The study could be helpful to design new reactive dyes with better affinity and selectivity for the cellulose fiber.

## 1. Introduction

Cellulose/cotton fiber is the backbone of the textile industry. The most preferable dyes for printing the cellulose/cotton are reactive dyes. These dyes have been used for over fifty years on an industrial scale. Rattee and Stephens invented the first reactive dye in 1954 which became commercially available in 1956 [1–3]. Reactive dyes have complicated chemical structures which form covalent bonds between reactive groups of the cellulose and activated functional groups of the dye molecules. Reactive dyes are the most common dyes because of many advantages such as operating under mild conditions, stable structures and bright colors [4,5]. The main features of these dyes are to interact chemically with cellulosic fiber as well as maintain quality during washing, last on the fabric for a longer duration and preserve the fixation value. All these features play an important role for the superiority of a reactive dye towards other types of dyes [6,7]. These dyes contain a reactive group, either a halo-heterocyclic or an activated double bond-containing chromophoric group that allows them to react directly with the surface of the cellulose fiber in order to make a chemical bond. The quality of a good dye actually depends on the interaction of the chemically-bound functional groups, e.g., sulfonic, hydroxyl, azo, carbonyl, and chloro groups of the dye with the molecules of cellulose in normal conditions, staying bonded during washing and being resistant to being washed out from the fabric. Some reactive dyes have a low potential for cellulose as compared to the acid dyes while most of the dyes have high selectivity of direct dyes. Therefore, the alkaline condition is suitable for the reaction of red reactive dyes with cellulose as well as for the adsorption and diffusion process.

**Figure 1:**
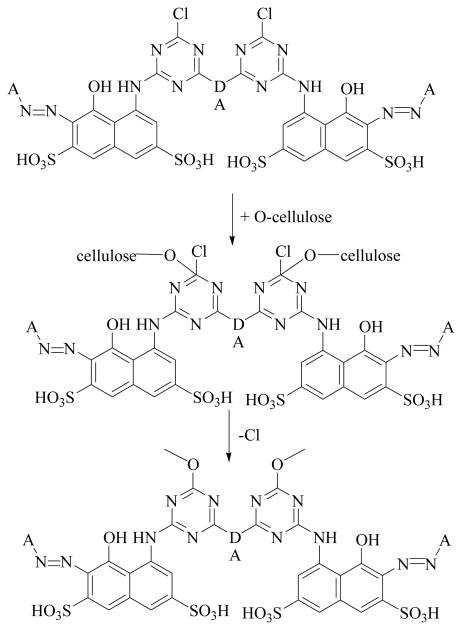
General mechanism of the reactive dye-cellulose interaction.

*Mechanism of Reactive Dyes*: Reactive dyes are nitrogen-containing heterocyclic rings bearing halogen substituents, therefore, undergo nucleophilic substitution reaction with the cellulose fiber. The heteroatom activates the system for nucleophilic attack due to its electro-negativity. The attacking nucleophile can be either a cellulose anion or a hydroxyl ion. This leads to fixation on the fabric after hydrolysis occurs of the reactive dye as shown in Figure 1. In addition, it is also important for a dye molecule to have a high dye-fabric covalent fixation value (F). This value is helpful to measure the dye affinity at the substrate or the amount of dye removed after the “soaping off process”. A high fixation value is helpful to reduce the time limit which is required for the dyeing process and also has an effect on the cost [8]. Besides, it is a measure of the extent of covalent bonding with the cellulose [9]. Dye fixation to cellulose fabric is triggered by acid-binding agents such as sodium hydroxide (NaOH), potassium hydroxide (KOH), sodium carbonate (Na_2_CO_3_), sodium bicarbonate (NaHCO_3_), potassium carbonate (K_2_CO_3_), sodium formate (HCOONa), sodiumdihydrogen phosphate (NaH_2_PO_4_) or disodium hydrogen phosphate (Na_2_HPO_4_). Higher fixation efficiency can be achieved by increasing reactive sites at the dye molecule by introducing two or more activating groups which react with the fabric. In this way, unfixed dye concentrations will reduce in the dye bath after dyeing and it becomes more economical and environmentally friendly.

The main industrial problem with commercially available dyes is the left over dyes in dye baths which is harmful for the environment; this factor is measured by dye-bath exhaustion (E) phenomena. The higher the exhaustion value, the lower the free dye remaining in the effluent after completion of the dyeing process. Numerous reports have been published to avoid the hazardous environmental effect during cellulose printing [10,11]. Therefore, our aim is to select those dyes which do not have a harmful effluent for the environment [12–18]. An optimal dye structure has the ability to react quantitatively with the cellulose fiber resulting in a dye-free bath. The dimmer form of reactive dyes allows to achieve excellent exhaustion values for printing the cellulose fiber [19].

In this study, the reactive dyes consist of the category of red reactive dyes known as azo dyes [20]. Dyeing by reactive dyes does not cause as many environmental problems as others [6,21]. Reactive dyes give equivalent wet-fastness, excellent properties and are widely used in textile industry. However, it is important to obtain good color which shows an absorbance of known concentration *i.e.*, an absorbtivity value (έ) of dm^3^/mol/cm × 10^4^.

Computational analysis can play an important role in selecting the binding nature of dyes with the cellulose by applying different methods in a minimum time frame. Dye-fabric interactions and receptor-ligand interactions have more or less similar concept. One of the most useful approache for the prediction of different properties initiate from the molecular structural information is known as the three-dimensional quantitative structure property relationships (3D-QSPR) [22,23]. 3D-QSPR studies are certainly of great importance in different branches of chemistry including medicinal chemistry, pharmaceutical chemistry and drug discovery [24,25]. This technique leads to locate the close relationship between bulk properties of compounds and their molecular structure which provides connection between the macroscopic and the microscopic properties of matter. Once the model has been developed then it can be utilized to predict properties of unknown compounds. A major step in constructing a 3D-QSPR model is to find a set of molecular descriptors that represent variation in the structural properties of the molecules. So far, a wide variety of descriptors have been reported in 3D-QSPR analysis [26–31]

In this project for the first time 3D-QSPR studies were applied on red reactive dyes (two different datasets) by using CoMFA protocol [32]. Basically, the interactions of various dye including the red reactive dyes with a cellulose fabric is a complex physicochemical process governed by specific features and the nature of the dye molecule. In CoMFA the interaction of dye-cellulose is affected by various factors including electrostatic and steric fields and the available experimental data. The aim of this study is to develop a predictive CoMFA model that correlates the absorbtivity of dyes with the modification of molecular structures. 3D-QSPR modeling tools have been used over the last two decades and considered as a most reliable method to understand the structural requirements of any property with interacting properties. Absorbtivity is the property considered for the current work which demonstrates how much the dye interacts with the cellulose fiber with low dye-bath exhaustion (E) and a high fixation value.

## 2. Computational Methodology

### 2.1. Molecular Modeling

All molecular modeling methods were performed using Sybyl7.3 [33] on a Genuine Intel^®^ Xeon (TM) 3.0 GHz dual core processor running under open SuSe Linux 11.0 environment. Initially, all structures were built by the Chem-Draw [34] and then converted into corresponding 3D structures using babel-2.1.1 [35].

### 2.2. Selection of Molecules

#### 2.2.1. Current Study Deals With The Two Different Data Sets

*Dataset I:* A set of thirteen compounds were retrieved from the literature reported by the *J Paluszkiewicz et al.* (dye-codes JP-1 to JP-13). They synthesized red reactive dyes which are derivatives of 1-amine-8-hydroxynaphthalene-3, 6-disulfonic acid [36].
*Dataset II:* Another dataset containing thirteen compounds was provided by Sitara *et al*. [37] with dye-codes (IS-14 to IS-26).

The complete dataset of twenty six compounds with their absorbtivity (ɛ) dm^3^/mol/cm are reported in Table 1. The selection of the training and test sets is based on random approach. Twenty-three (23) compounds were selected for the training set and the remaining three compounds were studied under the test set.

### 2.3. Dataset Preparation for the Alignment

The 3D structures of dyes were corrected with the help of Sybyl7.3 and the compound properties checked by Filter package of OpenEye. The geometry of all the compounds was minimized by the conjugate gradient method using Tripos force field [38] with 1000 iteration. After minimization, the maximum conformations were generated by Omega and the best conformation of each compound was selected for further work strategy. Gasteiger Hückel [39], am1bcc [40] and [41] charge methods were utilized for the calculation and these charges were applied on the whole dataset by molcharge utility of the OpenEye Quacpac program [42].

### 2.4. Structural Alignment

Alignment is one of the most significant steps for CoMFA studies. The 3D structures of the reactive dye molecules were aligned according to a suitable conformational template which showed higher interactive ability with the cellulose. In this case there are no previously reported data available for these reactive dyes which indicate the higher potency of structure within the dataset. Therefore, highest absorbtivity compound was considered as a template molecule. For the current work **JP-12** was considered as a most reactive dye showed highest absorbtivity 6.32 dm^3^/mol/cm × 10^4^ at 515.5 λ_max_.

### 2.5. Comparative Molecular Field Analyses (CoMFA) Study

All Comparative Molecular Field Analyses (CoMFA) [43–46] was performed using Sybyl7.3. Compounds were placed at the 3D lattice by following default setting of the CoMFA procedure with a 2.0 Å grid spacing. A sp^3^ carbon atom with +1 charge was employed to probe the steric (Lennard-Jones) and electrostatic (Coulombic) field energies. The cutoff interaction energy ± 30 kcal/mol was applied on both the CoMFA fields. These fields were generated automatically and scaled by the CoMFA-STD method. An attenuation factor of 0.1 was used.

### 2.6. Partial Least Square Analysis (PLS)

The Partial Least Squares (PLS) method [47–49] was used to construct and validate the 3D-QSPR model. The CoMFA descriptors served as independent variables and property values as dependant variables in PLS. This expresses the absorbtivity in terms of linear combinations of the CoMFA model generated as steric and electrostatic field.

The predicative potential of the CoMFA protocol was analyzed by the “leave-one-out” (LOO) [50] cross-validated analysis method, in which one compound is excluded systematically from the dataset and its property predicted using the model derived from the rest of the compounds. Column filtering was set at 2.0 kcal/mol for analysis which reduced the noise level. The cross-validated q^2^ value that resulted in a minimal number of components and the lowest standard error of prediction (SEP) was accepted for further study. The results obtained from the leave-one-out procedure yields an optimal number of components (ONC) which is associated by the non-validation PLS analysis. The PLS analysis was repeated for the non cross validation with the ONC to get the final model. In the end, the CoMFA results were graphically interpreted by field contribution maps using the field type “stdev.coeff”.

## 3. Results and Discussion

**Figure 2:**
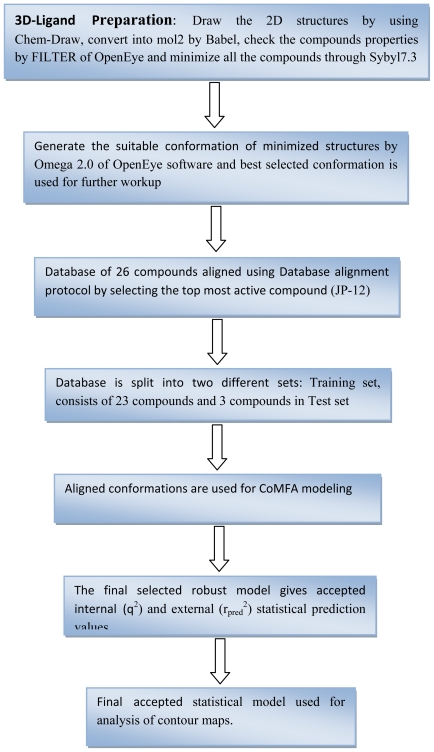
Work Flow Scheme for CoMFA Modeling.

From the literature survey it is quite clear that 3D-QSPR predicted a reliable model which could help to design new reactive dyes according to the modification of the developed features. In the current study we reported the application of the CoMFA modeling on red reactive dyes. CoMFA has been applied to derive the relationship between the structural modification and absorbtivity which give the direct indication of the influence on the dimmer form of red reactive dyes binding on cellulose. The work flow strategy is represented in Figure 2.

### 3.1. Selectivity Profile

The current work deals with dimmers of red reactive dyes. The reason for the selection of the dimmer form is that the monomer has low affinity with low soluble potential with cellulose fabric, as well as a less interactive functional group. However, on replacement of the dimmer forms, drastic changes were observed in the affinity of the reactive dyes with the cellulose structure. Therefore, we selected the dimmer series of the reactive dyes for the 3D-QSPR studies to predict the model which could help us to modify their structures. Finally, the results demonstrate that the outstanding modified structural features help to get more interaction of reactive dyes with the cellulose fiber.

### 3.2. Alignment Protocol with Different Charges

**Figure 3:**
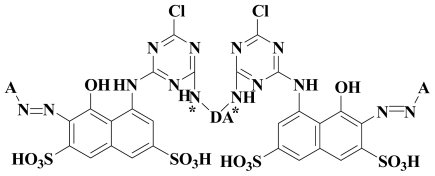
The most active dye molecule *JP-12* is used as a template and the selected atom involved in the alignment is shown by the asteric (*).

**Figure 4:**
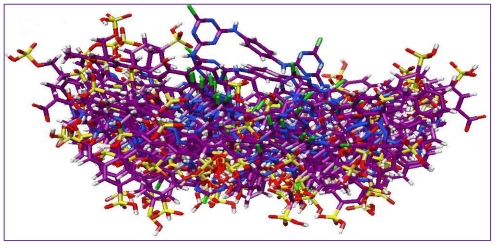
Structural alignment of all red reactive dyes by the database alignment method using the most active compound *JP-12* as template.

The common skeleton shown in Figure 3 was selected for the alignment and the rest of the molecules were aligned on the selected core structure, using the data base alignment method of Sybyl7.3. The aligned compounds were depicted in Figure 4. To get the best CoMFA model, three different charges methods were applied. After unsuccessful results by Gasteiger Hückel and am1bcc charge methods (data not shown), we considered another method for the study, known as MMFF94. By applying the MMFF94 charges and shuffling protocol on the training and test sets, we obtained good statistical results, as shown in Table 2.

### 3.3. Statistics of CoMFA Model

Twenty three (23) compounds were selected for the training set out of the total twenty-six (26) compounds to develop the CoMFA model. The remaining three compounds were utilized as test set for the external validation of the CoMFA model. The PLS method gives satisfactory results in term of q^2^ and r^2^ values, exhibiting the robustness of the developed model. The outcome of the PLS analysis is depicted in Table 3. The results indicated that leave one out cross validated q^2^ = 0.529 and the non cross validated r^2^ = 0.989. The electrostatic field contribution is assumed to be to some extent dominant as compared to the steric field, but in the broad spectrum, both fields have similar level of strength in field contribution.

### 3.4. 3D Analysis of the CoMFA

One of the most interesting and informative features of the CoMFA modeling is the visualization of the results as 3D coefficient contour plots. The contour maps were generated as scalar products of coefficients associated with each CoMFA column. The regions of having scaled coefficients greater than 80% (favored) or less than 20% (disfavored). The two fields of CoMFA models for the analysis based on the database alignment were presented as contour plots. The colored polyhedral in the map surrounded all lattice points where the 3D-QSPR is strongly associated with the changes occurred in the compounds field values with respect to absorbtivity potential.

**Figure 5:**
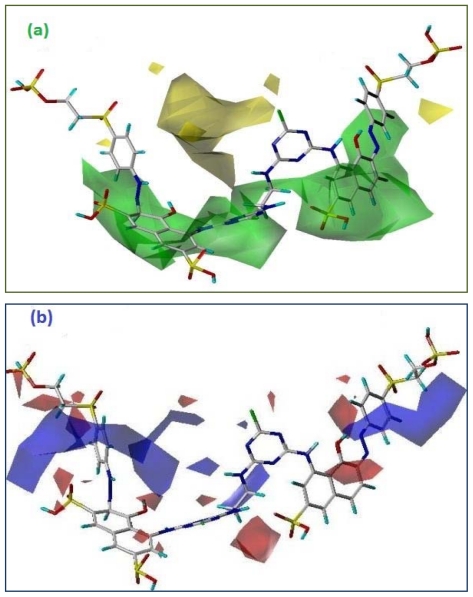
(**a**) CoMFA stDev*Coeff contour map based on the most active compound *JP-12* (Graphical representation of compound is displayed in sticks, carbon atom in gray with default elemental color). Steric fields: Favored for bulky groups (green) and disfavored for bulky group (yellow); (**b**) CoMFA stDev*Coeff contour map based on the most active compound *JP-12* (Graphical representation of the compound is displayed in sticks, carbon atom in gray with default elemental color). Electrostatic fields: Favored for negative group (red) and disfavored for negative group (blue).

Figure 5 (a and b) depict the steric and electrostatic contour maps of CoMFA (StDev*Coeff) model. The contour maps of electrostatic and steric contributions show useful potential in the design of new reactive dyes. To aid in the visualization, contour maps surround the template molecule JP-12 as a reference compound due to highest absorbtivity which indicates high affinity with the cotton fabric.

### 3.5. Steric Fields

The CoMFA contour plots in Figure 5(a) shows favorable regions in green color where increased steric function is associated with enhanced property function level and yellow region is dis-favorable and points out the increased steric bulk which is associated with decreased affinity of the dye molecule with cellulose. One large green polyhedron covers most of the bridging area “DA” of the reference compound. The “DA” group of the reference compound is an ethyl residue which has close contact with the green isopleths indicating that if a more bulky alkyl chain attaches, it could be beneficial in increasing the significant dye-cellulose interaction. If we compare JP-12 with JP-6, the molar absorbtivity is decreased, while both dyes have the same “DA” group. The reason for this lower value might be due to the “A” groups. In JP-12 a sulfonic group is attached which is responsible for better interaction with the positive part of the cellulose structure. A similar phenomenon occurs for JP-10 and IS-15: the absorbtivity decreases although both have the same bridging moiety and are depicted with green isopleths; here the side chain moieties play an important role for increasing the property profile level. Dye structures JP-8, JP-9, JP-11 and IS-17 have a sulfonic side chain “A” group; based on this group they have a high potency towards the interaction of cellulose fiber with these reactive dyes.

Several regions of yellow contours were found near the “A” groups, especially at the phenyl-based bridging residues with different attachments at different positions (*ortho, para and meta*) which indicates the significant decrease in the adsorption values of these dyes structures. Therefore, dye structures JP-1, JP-5, IS-18-19, IS-21 and IS-26 have a low activity profile. If we compare the compound IS-19 with IS-16, an increase in activity is observed, although both have a phenyl group at the “DA” moiety, so, ultimately our hypothesis of the sulfonic group is supported here.

### 3.6. Electrostatic Fields

The CoMFA electrostatic contour plot is displayed in Figure 5(b) with a 50.3% field contribution; it holds an important position in the current predicted model by CoMFA. Blue contour indicates the region where the negative potential is unfavorable for the increment in absorbtivity of the dye structure, whereas the red areas have a negative potential that are favorable for the improvement of the affinity of the dyes with cellulose. Electropositive favored and disfavored charge cut-off energies were also pinched at 90% and 10% of the dye structure.

The three large blue isopleths encompass the red reactive dye due to the nitrogen moiety which means that these regions are interacting well with the negative part of the cellulose. In the current work, the presence of nitrogen is conserved for all the molecules which is necessary for the dye structure, therefore, the red contour demonstrates better suggestions for the variation in the dataset.

Several red contours were found near the negative charge moieties mainly because of the hydroxyl and sulfonic groups. The compounds bearing the sulfonic group have the higher absorbtivity as compared to the rest of the groups, and as a result, JP-7, JP-9, JP-12, IS-15 and IS-16 show good absorbtivity values.

### 3.7. Validation of the 3D-QSPR Model

Three selected compounds were used as the test set to verify the constructed CoMFA model. The calculated results are listed in Table 2. The predicted absorbtivity for the CoMFA model are in good agreement with the experimental data, and in a statistically tolerable range, with the correlation coefficient of r^2^ = 0.797. The test results indicated that the CoMFA model is reliable and has good predictive ability.

## 4. Conclusion

In this study, the computational technique 3D-QSPR was applied to find the effects of structural modification of red reactive dyes and the binding mechanism with the cellulose fiber. Our study demonstrated that the CoMFA method is suitable and reliable for the prediction of the relation between structural features of red reactive dye and its absorbtivity. A robust CoMFA model was obtained with a high predictive performance for the red reactive dye.

Electrostatic and steric interactions are the most important features in reactive red dye-cellulose binding interaction. According to our results both fields play important roles for the current set of dyes. The steric field has drawn our attention towards the bulkiness; if we could increase the alkyl chain of the bridging moiety then it would give a more significant interaction. CoMFA studies concerned with the contribution of electrostatic fields, demonstrate that the positive charges in the dye molecule favor the dye adsorption on cellulose. Those molecules which have sulfonic groups, have high absorbtivity values because this moiety helps to solubilize the dye molecule inside the fabric. The information obtained from the 3D-QSPR model may provide a tool for predicting the affinity of unknown structures prior to its synthesis, which could be optimal for cellulose fiber as well as being non-toxic for the environment.

## Figures and Tables

**Table 1:** Red reactive dye structures and their absorbtivities (dm^3^/mol/cm × 10^4^).

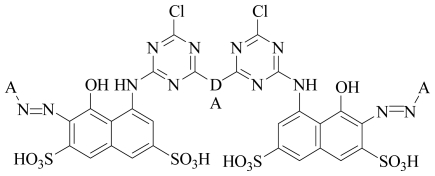				
S. No.	Dye Code	Group “A “	Bridging Moiety “DA”	Absorbtivity dm^3^/mol/cm × 10^4^
1	JP-1	Anthranilic acid	1,4-Phenylenediamine	3.47
2	JP-2	Anthranilic acid	1,2-Phenylenediamine	5.37
3	JP-3	Anthranilic acid	1,3 - Phenylen diamine	5.30
4	JP-4	Anthranilic acid	Diaminestilbene-2,2′-disulfonic acid	4.29
5	JP-5	Anthranilic acid	4,4′-Diaminebenzoanilide	3.74
6	JP-6	Anthranilic acid	Ethylene-1,2-diamine	4.94
7	JP-7	4-*β*-Sulphatoetyl-sulfonyl aniline	1,4-Phenylenediamine	5.43
8	JP-8	4-*β*-Sulphatoetyl-sulfonyl aniline	1,2-Phenylenediamine	6.21
9	JP-9	4- *β*-Sulphatoetyl-sulfonyl aniline	1,3-Phenylenediamine	5.81
10	JP-10	4- *β*-Sulphatoetyl-sulfonyl aniline	Diaminestilbene-2,2′-disulfonic acid	5.49
11	JP-11	4-*β*-Sulphatoetyl-sulfonyl aniline	4,4′-diaminebenzoanilide	4.95
12	JP-12	4-*β*-Sulphatoetyl-sulfonyl aniline	Ethylene-1,2-diamine	6.32
13	JP-13	Anrthanilic acid	1,4-Phenylenediamine	4.67
14	IS-14	Aniline	Diaminestilbene-2,2′-disulfonic acid	2.95
15	IS-15	Benzene-m-aminosulfonic acid	Diaminestilbene-2,2′-disulfonic acid	1.85
16	IS-16	Benzene-m-aminosulfonic acid	1,4-Phenylenediamine	0.71
17	IS-17	Benzene-p-aminosulfonic acid	1,4-Phenylenediamine	1.95
18	IS-18	Aniline	1,4-Phenylenediamine	0.37
19	IS-19	*p*-Toluidine	1,4-Phenylenediamine	0.25
20	IS-20	p-Toluidine	Diaminestilbene-2,2′-disulfonic acid	1.92
21	IS-21	*p*-Toluidine	1,3-Phenylenediamine	0.47
22	IS-22	*p*-Nitroaniline	1,4-Phenylenediamine	2.44
23	IS-23	*p*-Nitroaniline	1,3 - Phenylenediamine	1.74
24	IS-24	*m*-Toluidine	1,4 - Phenylenediamine	0.47
25	IS-25	*m*-Toluidine	1,3-Phenylenediamine	1.40
26	IS-26	*m*-Toluidine	Diaminestilbene-2,2′-disulfonic acid	2.32

**Table 2:** Actual and predicted absorbtivity of red reactive dyes of training and test sets.

Compounds	Experimental absorbtivity	Predicted absorbtivity by CoMFA
***Training set***		
JP-02	5.37	5.38
JP-03	5.30	5.46
JP-04	4.29	4.18
JP-05	3.74	3.67
JP-06	4.94	4.91
JP-07	5.43	5.66
JP-08	6.21	6.24
JP-09	5.81	5.85
JP-10	5.49	5.37
JP-12	6.32	6.51
JP-13	4.67	4.35
IS-14	2.95	2.52
IS-15	1.85	1.95
IS-16	0.71	0.67
IS-17	1.95	2.02
IS-18	0.37	0.43
IS-19	0.25	0.31
IS-20	1.92	1.35
IS-22	0.47	0.75
IS-23	2.44	2.41
IS-24	0.47	1.08
IS-25	1.4	1.39
**Test set**		
JP-01	3.47	5.40
JP-11	4.95	4.37
IS-21	0.47	2.18

**Table 3:** Statistical results for red reactive dyes extracted by CoMFA analysis.

Parameters	CoMFA
a*q^2^*	0.509
bONC	03
cSEP	1.567
dSEE	0.257
*F*-test ratio	591.27
e*r^2^*	0.989
f*r^2^_pred_*	0.797

**Field contribution**	**Percentage (%)**

Steric	49.7
Electrostatic	50.3

a Cross-validated correlation coefficient (q^2^);

b Optimum number of components (ONC);

c Standard error of prediction (SEP);

d Standard error of estimate (SEE);

e Conventional correlation coefficient (r^2^);

f Correlation coefficient (r^2^ _pred_).
